# Clinicopathologic Characteristics and Long-Term Outcome of Gastric Cancer Patients with Family History: Seven-Year Follow-Up Study for Korean Health Check-Up Subjects

**DOI:** 10.1155/2020/4028136

**Published:** 2020-10-22

**Authors:** Jooyoung Lee, Su Jin Chung, Ji Min Choi, Yoo Min Han, Joo Sung Kim

**Affiliations:** ^1^Department of Internal Medicine and Healthcare Research Institute, Seoul National University Hospital Healthcare System Gangnam Center, Seoul, Republic of Korea; ^2^Department of International Medicine and Liver Research Institute, Seoul National University College of Medicine, Seoul, Republic of Korea

## Abstract

**Methods:**

This study was conducted on asymptomatic healthy individuals who underwent upper gastrointestinal endoscopy for the purpose of GC screening. Patients who were diagnosed with GC between October 2003 and December 2013 at Seoul National University Hospital Healthcare System Gangnam Center were identified. Demographic and clinicopathologic characteristics were compared between the groups with and without FHx of GC. Overall survival (OS) and recurrence-free survival (RFS) were assessed as primary outcomes.

**Results:**

There were no significant differences in tumor characteristics according to FHx of GC. However, preexisting adenoma was more frequent in patients with FHx than in those without FHx (14.5% vs. 6.3%, *p* = 0.035). The proportion of patients with microsatellite instability (MSI) was also higher in groups with FHx of GC (43.2% vs. 13.2%, *p* = 0.006). *Helicobacter pylori* infection rates of patients with FHx of GC tended to be higher although not significant (70.5% vs. 61.3%, *p* = 0.188). However, OS and RFS at 5 years of the GC patients with FHx were not significantly different from those of patients without FHx.

**Conclusion:**

Preexisting adenoma and GC with MSI are more common in patients with FHx of GC than in those without. There were no significant differences in the survival rate according to FHx.

## 1. Introduction

The incidence of gastric cancer (GC) has declined significantly worldwide over the past half-century and is possibly attributable to economic development, improved sanitation, and decreased *Helicobacter pylori* infection rates because of the expansion of eradication [1]. Nevertheless, GC remains a global health problem as the fifth leading cancer and the third most common cause of cancer-related deaths worldwide. Newly diagnosed GC cases were estimated at 1,034,000 in 2018, representing 5.7% of all newly diagnosed malignancies and accounting for 8.2% of cancer-related deaths [2]. South Korea is one of the high prevalence regions of GC and approximately 26,000 new GC cases and about 7,100 GC-related deaths were reported in 2018 [3]. Therefore, identifying risk factors for GC and managing high-risk individuals through active prevention and early detection strategies may be important in areas with high prevalence of GC, to reduce the socioeconomic burden caused by the disease.

Environmental factors such as *H*. *pylori* infection, cigarette smoking, and excessive salt intake are known to be related to the development of GC [4, 5]. In addition, family history (FHx) of GC has been reported to be a strong risk factor of GC; approximately 10%–20% of GC patients have FHx of the disease [6, 7]. Genetic predisposition, intrafamilial transmission of *H*. *pylori* infection, and shared dietary habits within family members may result in the increased familial aggregation of GC.

Several studies have investigated the clinicopathologic differences between GC patients with and without FHx of GC; however, conflicting results have been reported. There are also debates regarding the impact of FHx on disease progression and prognosis. Therefore, we aimed to investigate the clinicopathologic characteristics of GC according to the FHx of GC and to analyze the effect of FHx on long-term outcomes.

## 2. Material and Methods

### 2.1. Study Population

Approximately 20,000 people every year visit the Seoul National University Hospital (SNUH) Healthcare System Gangnam Center in Seoul, South Korea, for comprehensive medical check-ups. The Health and Prevention Enhancement (H-PEACE) study is a cohort that summarizes and integrates all results of these health check-ups [8]. The H-PEACE study includes not only data widely used in medical check-ups but also data from high-quality advanced examinations for prediction of preclinical stages of noncommunicable diseases, including malignancies and metabolic disease in an average-risk healthy population in Korea. The GC cohort is a subcohort of the H-PEACE study, and we have obtained all medical records through this study.

Subjects who underwent upper gastrointestinal (GI) endoscopy as a screening test for GC between October 2003 and December 2013 were potentially eligible for our retrospective cohort. The inclusion criteria for enrollment of this study were as follows: (1) diagnosis histologically confirmed as GC; (2) complete staging evaluation for GC, including radiologic imaging and pathologic examinations; (3) complete comprehensive questionnaire regarding risk factors of GC, including FHx of GC; and (4) at least four visits according to the follow-up schedule after GC treatment.

This study protocol was approved by the Institutional Review Board (IRB) of SNUH (H-1803-049-928) and was conducted in accordance with the Declaration of Helsinki. Informed consent was waived by the SNUH IRB because of the retrospective study design.

### 2.2. Demographic and Clinicopathologic Findings

All subjects were requested to complete a self-administered, structured questionnaire including FHx of GC, cigarette smoking, and alcohol consumption status. FHx of GC was considered to be present when there was at least one first-degree relative diagnosed with GC. Current smoker was defined when the subject smoked at least one cigarette per day for the previous 12 months. When the subject drank >140 g/week of alcohol, we regarded this as excessive alcohol consumption. The interval between the time of upper GI endoscopy most recently confirming normal findings and the time of cancer diagnosis was also analyzed.

Tumor size, location, differentiation, Lauren's classification, depth of invasion, lymph node metastasis, distant organ metastasis, and multiplicity of tumor and stage of diagnosis were recorded. Preexisting adenoma was regarded as present when adenomatous components at the margin of GCs were observed on pathologic examination. Microsatellite instability (MSI) was defined at the following loci according to the National Institutes of Health guidelines: BAT25, BAT26, D2S123, D5S346, and D1S250. We assigned tumors as MSI-high (MSI-H) when two or more markers showed instability and as MSI-low (MSI-L) when one marker showed instability. Microsatellite stable status (MSS) was assigned when none of the markers was unstable [9]. *H*. *pylori* infection status was determined by histologic evaluation, rapid urease test, and serum *H*. *pylori* IgG antibody test. Serologic tests for *H*. *pylori* infection were performed on the day of the endoscopic examination using a commercially available enzyme-linked immunosorbent assay [10]. Atrophic gastritis (AG) was diagnosed endoscopically if a pale gastric mucosa with prominent submucosal vascularity was noted. Endoscopic features with mucosal nodularity and multiple whitish plaques were regarded as intestinal metaplasia (IM).

Curative treatment methods were also reviewed and classified as endoscopic submucosal dissection (ESD) and surgery including subtotal gastrectomy and total gastrectomy. The indications for ESD were as follows: (1) differentiated adenocarcinoma, (2) lesions≤2 cm in diameter on endoscopic estimation, and (3) no evidence of submucosal invasion and lymph node/distant organ metastasis on endoscopic ultrasonography and/or abdominal computed tomography (CT). Surgery was performed in all cases outside of this indication.

### 2.3. Follow-Up Schedule

Patients who were treated by ESD were scheduled to follow-up by endoscopic examination at 3, 6, and 12 months after ESD and annually thereafter [11, 12]. Postoperative follow-up was performed every 3 months for the first two years and then every 6 months from 2 to 5 years after surgery. Ultrasonography, abdominal CT, and endoscopy were performed once or twice a year until 2 years postoperatively and annually thereafter [13].

### 2.4. Statistical Analysis

Continuous variables were presented as means and standard deviations. Categorical variables were presented as numbers and percentages. Comparison of demographic and clinicopathologic findings were performed using the Student *t*-test for continuous variables and the chi-square test for categorical variables. The primary outcomes for investigating long-term prognosis were overall survival (OS) and recurrence-free survival (RFS). OS was defined as the time from primary curative treatment to death resulting from any cause. RFS was defined as the time from primary curative treatment to tumor recurrence and death with evidence of GC recurrence. These survival rates were calculated using the Kaplan–Meier method with the log-rank test. Data analysis was performed using SPSS version 25.0 (SPSS Inc., Chicago, IL, USA), and a *p* value of <0.05 was considered statistically significant.

## 3. Results

### 3.1. Study Population

**Figure 1:**
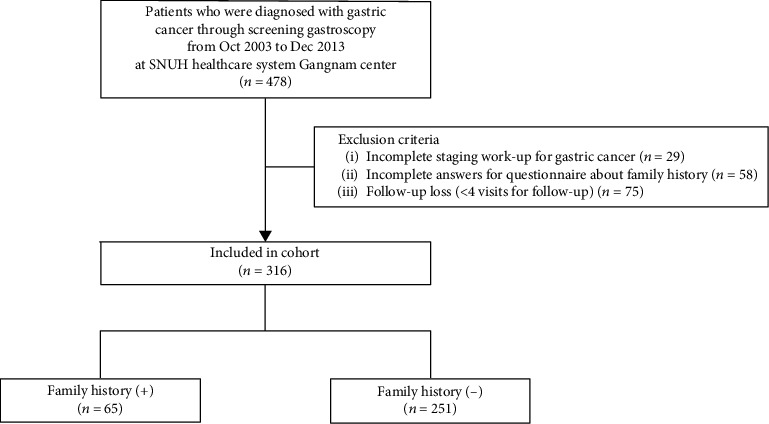
Flow chart of enrollment of the study population.

A total of 478 GC patients were screened from our cohort. Of these, 29 patients who had not completed the cancer staging evaluation, 58 patients who had no information regarding FHx of GC, and 75 patients who were lost to follow-up were excluded. Therefore, 316 patients were included in the final analysis. Of these patients, 65 (20.6%) had a family history of GC (Figure 1). Fifty-six patients had a family history in first-degree relatives, and 9 patients had both first- and second-degree family history of GC. A total of 263 patients (83.2%) underwent follow-up evaluation according to their planned follow-up schedule and visited outpatient clinic at SNUH regularly at least 5 years after the diagnosis. The proportion of patients with FHx who followed up for more than 5 years was 86.1%, and 82.4% of the patients without family history were followed up for more than 5 years.

### 3.2. Demographic and Clinicopathologic Characteristics

The demographic findings of the study population are summarized in Table 1. The proportion of patients aged 50–59 was the highest in both groups regardless of FHx of GC. Although the difference was statistically insignificant, the age of patients with FHx of GC at the cancer diagnosis was higher than that of patients without FHx of GC (58.6 ± 9.3 years vs. 55.9 ± 10.7 years, *p* = 0.059). Males were 71.8% of the total cohort, and there was no significant difference in gender between groups. Health-related behaviors, including cigarette smoking, alcohol consumption, and salt intake were not significantly different between groups. When the interval of upper GI endoscopy was compared, patients with family history of GC were more likely to have intervals of less than two years between the time of upper GI endoscopy confirming normal findings and the time of cancer diagnosis (33.8% vs 21.9%, p =0.046).

Comparison of the clinicopathologic findings between the groups are presented in Table 2. Pathologic examinations showed no significant differences in histopathological findings, including tumor location and size, differentiation, Lauren's classification, depth of invasion, lymph node metastasis, and multiplicity of tumors between the groups. However, preexisting adenoma was found more frequently in patients with FHx of GC than in those without FHx (14.5% vs. 6.3%, *p* = 0.035). MSI was evaluated in 136 of the patients who underwent ESD or surgery. The proportion of patients with MSI was significantly higher in groups with family history of GC (43.2% vs. 13.2%, *p* = 0.006). Although statistically insignificant, *H*. *pylori* infection rate of patients with FHx of GC tended to be higher than that of patients without FHx (70.5% vs. 61.3%, *p* = 0.188). Presence of endoscopic AG/IM did not show any significant difference between the groups; however, IM was more common in patients with FHx of GC. Overall, most GC patients (*N* = 260, 82.3%) had stage I GC, and fourteen (4.4%) had stage IV at diagnosis. There was no significant difference in tumor stage between groups. Ninety-six (30.3%) patients received ESD for treatment of GC, and surgery was performed in 206 patients (65.2%). The remaining 14 patients received palliative chemotherapy as the first treatment without ESD or surgery.

### 3.3. Comparison between Gastric Cancer-Related Survival Rates

**Figure 2:**
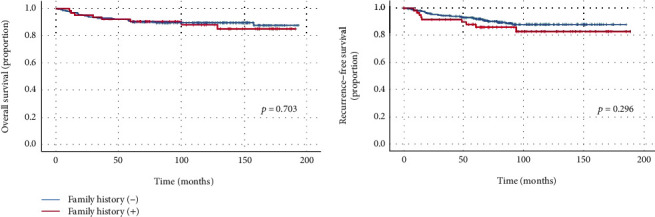
Survival curves of patients with family history and without family history.

During the median follow-up duration of 80.8 months (range, 4.8–187.4 months), 34 patients (10.8%) died. Of these, 27 patients died of GC. Twenty-three patients (7.2%) had recurrence of GC, of which 13 had metachronous GC recurrence. The OS and RFS at 5 years of total study population were 90.6% and 91.1%, respectively. The OS at 5 years for patients with FHx of GC was 90.6%, not significantly different from that of patients without FHx of GC (*p* = 0.703, Figure 2). The RFS at 5 years of patients without FHx were numerically higher than those of patients with FHx of GC; however, there were no significant differences between the groups (92.2% vs 86.4%, *p* = 0.296 (Figure 2)).

**Figure 3:**
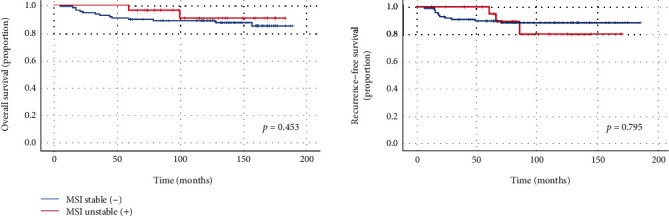
Survival curves of patients according to microsatellite instability (MSI) status.

The OS and RFS according to MSI status were assessed for the study population surveyed for MSI (Figure 3). When the subjects were divided into the MSI group and microstallite stable (MSS) group, the OS at 5 years of the MSI group was numerically higher than that of the MSS group regardless of GC FHx (96.3% vs. 89.8%, *p* = 0.453). The RFS at 5 years of the MSI group was also higher than that of the MSS group, though it was not statistically significant (95.0% vs. 89.8%, *p* = 0.795).

## 4. Discussion

In the present study, we investigated the clinicopathologic characteristics and long-term outcomes of GC according to the FHx of GC in a health screening cohort in a high-GC-prevalence region of Korea. There were no significant differences in the tumor characteristics or overall prognosis between GC patients with and without FHx of GC. However, the proportion of patients with preexisting adenoma and MSI were significantly higher in groups with FHx of GC.

Choi et al. detected preexisting adenoma in 15.6% of endoscopically resected early gastric cancer (EGC) cases and reported that EGC cases with preexisting adenoma showed a greater association with *H*. *pylori*-related chronic inflammation than those without preexisting adenoma [14]. According to the hypothesis of gastric carcinogenesis by Correa et al., it is widely accepted that chronic inflammation triggered by *H*. *pylori* infection develops into AG, IM, dysplasia, and finally adenocarcinoma [15]. The higher presence of preexisting adenoma in patients with FHx of GC could be explained by the higher *H*. *pylori* infection rate in the same group. Gastric adenoma is thought to be a precancerous lesion; the annual incidence of GC is 0.6% among patients with mild to moderate dysplasia and 6% among those with severe dysplasia [16]. Our results suggest that early detection of precancerous lesions through active screening for high-risk groups including those with FHx of GC may help prevent the development of GC.

The prevalence of MSI-high GC in Asians is commonly <10% of all GC cases and is lower than that shown in Western studies [17, 18]. Higher presentation of MSI in patients with FHx of GC is known based on several prior studies, and this was consistent with our findings [19–21]. MSI-high GC is known to be associated with intestinal-type histology, infrequent lymph node metastasis, and better overall prognosis [22–24]. Although statistically insignificant, the OS of patients with MSI was higher in our study than those without. However, since most of our study subjects were diagnosed with EGC with favorable prognosis, there is a limitation to clearly revealing the survival difference according to MSI status. The relationship between survival in GC patients and MSI has yet to be determined, and there was heterogeneity in the recent studies [25]. Further studies with a larger number of patients are needed.

There are some studies regarding the effect of FHx of GC on the long-term outcome of GC. A Korean study including 1,265 GC patients reported that survival rates of GC patients with FHx of GC did not significantly differ from those of patients without FHx of GC [26]. Another large-scale Korean study showed that first-degree FHx of GC was associated with improved survival after curative surgery in patients with stage III or IV GC [27]. A meta-analysis suggested that FHx of GC is associated with better survival of GC patients after curative surgery [28]. We found that there were no significant differences in OS and RFS at 5 years between the groups. This study was conducted on asymptomatic healthy individuals who underwent screening endoscopy in a single center. Therefore, most of the diagnosed tumors were early-stage GC (EGC of stage I), presenting a limitation in terms of the interpretation and generalization of our results, considering the characteristics of the study population.

The interval between the time of upper GI endoscopy confirming normal findings and the time of cancer diagnosis of patients with FHx of GC was less than 2 years, and this was much shorter than that of patients without FHx of GC. This result may reflect the health-related behaviors of individuals with risk factors for GC. A large cohort study using the Korean National Health and Nutrition Examination Survey 2005 database showed that GC relatives were significantly more likely to undergo GC screening than were non-GC relatives [29]. Considering the high compliance with screening tests of relatives of GC patients and the high prevalence of GC following the adenomacarcinoma sequence in patients with FHx of GC, it is advisable to recommend active endoscopy screening with a shorter interval for relatives of GC patients.

This study has several strengths. First, to the best of our knowledge, this is the first study to examine associations between FHx of GC and preexisting adenoma as a precancerous lesion. These results can be used to support the necessity of active surveillance in a high-risk subgroup of FHx of GC for the early detection of precancerous lesions and prevention of GC development. Our prior study also showed that intensive screening and surveillance may be useful for high-risk subpopulations with epidemiologic risk factors or premalignant lesions such as IM [30]. Second, we controlled for referral bias by enrolling individuals from a health check-up cohort representing the general population with an average risk. Third, we collected, organized, and qualified medical records from our H-PEACE study.

Several factors should be considered when interpreting the results of this study. First, because of the retrospective study design, *H*. *pylori* eradication history was not thoroughly investigated. It cannot be ruled out that the possible association between *H*. *pylori* infection status and FHx of GC was underestimated. Second, there is some possibility that information about the FHx of GC is inaccurate because it was based on a self-administered questionnaire. Nevertheless, consistency and reliability of the self-reported family history was validated by findings of previous studies [31, 32].

In conclusion, preexisting adenoma is more common in patients with FHx of GC than those without FHx of GC; GC with MSI is associated with FHx of GC. The survival of patients with FHx of GC did not significantly differ from that of those without family history. Early detection of precancerous lesions through active screening for high-risk groups with FHx of GC may help prevent the development of GC.

## Figures and Tables

**Table 1:** Demographic findings of study population.

	Family history of gastric cancer				*p* value
	Absent (*n* = 251)		Present (*n* = 65)		
	No.	%	No.	%	
Age (yr)					
≤39	20	8	2	3.1	0.439
40-49	42	16.7	8	12.3	
50-59	101	40.2	27	41.5	
60-69	62	24.7	18	27.7	
≥70	26	10.4	10	15.4	
Mean + SD	55.9 ± 10.7		58.6 ± 9.3		0.059
Gender					0.306
Male	177	70.5	50	76.9	
Female	74	29.5	15	23.1	
Smoking					0.432
Current	67	28.5	17	28.3	
Ex-smoker	83	35.3	26	43.3	
None	85	36.2	17	28.3	
Excess alcohol (>140 g/day)					0.167
Yes	112	47.5	35	57.4	
No	124	52.5	26	42.6	
Excess salt intake					0.839
Yes	148	63	38	64.4	
No	87	37	21	35.6	
EGD interval^∗^					0.046
<2 yr	55	21.9	22	33.8	
≥2 yr	196	78.1	43	66.2	

EGD: esophagogastroduodenoscopy; SD: standard deviation. ^∗^Interval between the time of upper GI endoscopy confirming normal findings and the time of cancer diagnosis.

**Table 2:** Clinicopathologic findings of gastric cancer patients.

	Family history of gastric cancer				*p* value
	Absent (*n* = 251)		Present (*n* = 65)		
	No.	%	No.	%	
Tumor location					0.17
Upper third	29	11.6	8	12.3	
Middle third	79	31.5	13	20	
Lower third	139	55.4	41	63.1	
All	4	1.6	3	4.6	
Tumor size (cm)					0.78
<1.0	46	18.3	11	16.9	
1.0-1.9	73	29.1	19	29.2	
2.0-2.9	41	16.3	14	21.5	
≥3.0	91	36.3	21	32.3	
Mean + SD	2.8 ± 2.3		2.8 ± 2.5		0.991
Differentiation					0.197
Differentiated	132	52.6	40	61.5	
Undifferentiated	119	47.4	25	38.5	
Laruen classification					0.491
Intestinal	133	57.1	39	61.9	
Diffuse or mixed	100	42.9	24	38.1	
Depth of invasion					0.756
pT1	193	76.9	49	75.4	
pT2	32	12.7	11	16.9	
pT3	13	5.2	3	4.6	
pT4	13	5.2	2	3.1	
Lymph node metastasis					0.235
pN0	199	79.3	56	86.2	
pN1	26	10.4	6	9.2	
pN2	15	6	0	0	
pN3	11	4.4	3	4.6	
Multiplicity of tumor					0.653
Solitary	239	95.2	61	93.8	
Multiple	12	4.8	4	6.2	
Stage					0.698
I	204	81.3	56	86.2	
II	28	11.2	4	6.2	
III	8	3.2	2	3.1	
IV	11	4.4	3	4.6	
Preexisting adenoma					0.035
Yes	15	6.3	9	14.5	
No	222	93.7	53	85.5	
Microsatellite instability					0.006
Stable	86	86.8	21	56.8	
Unstable, low (MSI-L)	6	6.1	7	18.9	
Unstable, high (MSI-H)	7	7.1	9	24.3	
Curative treatment method					0.538
ESD	78	32.6	18	28.6	
Surgery (STG+TG)	161	67.4	45	71.4	
H. pylori infection status					0.188
Yes	138	61.3	43	70.5	
No	87	38.7	18	29.5	
Atrophic gastritis					0.354
Yes	231	92	62	95.4	
No	20	8	3	4.6	
Intestinal metaplasia					0.113
Yes	137	53.8	44	67.7	
No	114	45.4	21	32.3

ESD: endoscopic submucosal dissection; *H*. *pylori*: Helicobacter pylori; SD: standard deviation; STG: subtotal gastrectomy; TG: total gastrectomy. Microsatellite instability was evaluated in 136 of the patients (43% of total study population) who underwent ESD or surgery.

## Data Availability

The data used to support the findings of this study are available within the manuscript.

## Abbreviations

GC: Gastric cancer
FHx: Family history
MSI: Microsatellite instability
OS: Overall survival
RFS: Recurrence-free survival.
